# Three-Dimensionally Ordered Macroporous ZnO Framework as Dual-Functional Sulfur Host for High-Efficiency Lithium–Sulfur Batteries

**DOI:** 10.3390/nano10112267

**Published:** 2020-11-16

**Authors:** Haisheng Han, Tong Wang, Yongguang Zhang, Arailym Nurpeissova, Zhumabay Bakenov

**Affiliations:** 1 School of Materials Science and Engineering, Hebei University of Technology, Tianjin 300130, China; 15022610664@139.com (H.H.); 13720007220@139.com (T.W.); 2 Tianjin Key Laboratory of Materials Laminating Fabrication and Interface Control Technology, Hebei University of Technology, Tianjin 300130, China; 3 Department of Chemical and Materials Engineering, National Laboratory Astana, Nazarbayev University, Nur-Sultan 010000, Kazakhstan; arailym.nurpeissova@nu.edu.kz (A.N.); zbakenov@nu.edu.kz (Z.B.)

**Keywords:** lithium–sulfur battery, three-dimensionally ordered macroporous ZnO, strong adsorption, fast redox kinetics

## Abstract

A three-dimensionally ordered macroporous ZnO (3DOM ZnO) framework was synthesized by a template method to serve as a sulfur host for lithium–sulfur batteries. The unique 3DOM structure along with an increased active surface area promotes faster and better electrolyte penetration accelerating ion/mass transfer. Moreover, ZnO as a polar metal oxide has a strong adsorption capacity for polysulfides, which makes the 3DOM ZnO framework an ideal immobilization agent and catalyst to inhibit the polysulfides shuttle effect and promote the redox reactions kinetics. As a result of the stated advantages, the S/3DOM ZnO composite delivered a high initial capacity of 1110 mAh g^−1^ and maintained a capacity of 991 mAh g^−1^ after 100 cycles at 0.2 C as a cathode in a lithium–sulfur battery. Even at a high C-rate of 3 C, the S/3DOM ZnO composite still provided a high capacity of 651 mAh g^−1^, as well as a high areal capacity (4.47 mAh cm^−2^) under high loading (5 mg cm^−2^).

## 1. Introduction

The lithium–sulfur (Li-S) battery has attracted extensive attention owing to its high theoretical capacity (1672 mAh g^−1^), high energy density (2600 Wh kg^−1^), inexpensiveness, and the environmental benignity of sulfur [1,2,3]. However, Li-S batteries have serious performance defects that limit any further and potential applications [4,5]. This is due to the insulation nature of sulfur, the dissolution of lithium polysulfides (LiPSs), and the formation of insoluble Li_2_S_2_/LiS_2_ during operation [6,7,8]. Various methods were adopted to improve the electrochemical properties of the Li-S battery including the addition of sulfur into a conductive matrix, modification of an interlayer on separator and design of novel electrolytes [9,10,11,12,13,14,15]. Among these methods, an application of carbon materials as a sulfur host has been proved to be effective in improving electrode conductivity and the confinement of LiPSs [16]. However, during the long-term cycling, the capacity of carbon materials decays seriously and the Coulombic efficiency is very low, which are mainly because non-polar carbon cannot provide strong enough physical limitations to reduce the dissolution of high-polar and ionic polysulfides [17].

In recent years, the matrix materials that have strong chemical interactions with polysulfides are becoming more and more promising for the purposes mentioned above. For example, a modified carbon substrate doped with nitrogen, sulfur, oxygen, and other polar heteroatoms is used to anchor polysulfides in the cathode region [18,19]. Another matrix material, the metal–organic framework (MOF), can effectively slow down the diffusion of polysulfides and show good cyclic stability due to the strong Lewis acid–base interactions with polysulfides [20,21,22]. In addition, various metal oxides, sulfides, and nitrides have also been used as sulfur reservoirs because of their strong interactions with polysulfides to improve the sulfur utilization and cycle stability [23,24,25,26,27,28]. However, the metal oxides always have low electrical conductivity and limited specific surface area, which inevitably reduces the ability to anchor polysulfides. In addition, the commonly used polar metal oxide materials, usually with irregular particle forms, provide a restricted active surface to absorb polysulfides [29]. Therefore, it is necessary to design a material with fast electronic channels connected through a high-porosity network to ensure sufficient space for the storage of active substances and adsorption of polysulfides to achieve high electrochemical utilization and promote redox reactions.

Among all possible metal oxide candidate materials, ZnO has attracted an immense amount of attention considering its environmental friendliness, polarity, and other advantages [30]. Herein, we prepared a three-dimensionally ordered macroporous ZnO (3DOM ZnO) framework with good interconnected large pores and high specific surface area. The ordered porous structure not only promotes the transportation of ions, electrons, and active substances, but also effectively restricts polysulfides. In addition, the increased active surface areas possess a strong chemical reaction and catalytic effect for polysulfides, which effectively inhibit the shutting of polysulfides and promote the kinetics of sulfur redox reaction. The electrochemical performance test results show that 3DOM ZnO possesses a high capacity, excellent cycle performance, and high rate performance, which are all remarkably better than those of traditional three-dimensional (3D) ZnO. This study will help to reveal the effect of the 3DOM structure on the electrochemical behavior of sulfur and provide a basis for the development of high-performance Li-S batteries.

## 2. Materials and Methods

### 2.1. Preparation of PMMA@Precursor

Polymethyl methacrylate (PMMA) nanospheres were synthesized by the traditional suspension polymerization method to fabricate templates [31]. To prepare PMMA@precursor, Zn(NO_3_)_3_∙6H_2_O (3.56 g) and citric acid (2.25 g) were added to anhydrous methanol (5 mL) and stirred for 1 h. Afterwards, fabricated PMMA (2.0 g) nanospheres were immersed in this solution and stirred for 4 h.

### 2.2. Preparation of 3DOM ZnO and 3D ZnO

The obtained solution was transferred to a vacuum box and degassed for 10 min to fill up the possible empty intervals in PMMA templates. Then, the PMMA@precursor was dried in an oven and calcined in argon for 3 h at 300 °C with the heating rate of 1 °C min^−1^. A 3DOM ZnO framework was obtained by further heating the obtained material at 600 °C for another 3 h with a heating rate of 1 °C min^−1^ in the air. After gradual cooling to room temperature, the yellow powder was collected. Meanwhile, 3D ZnO was obtained by increasing the temperature further to 800 °C. 

### 2.3. Preparation of S/3DOM ZnO

3DOM ZnO and sulfur were evenly mixed at a ratio of 1:2 by weight. Then, the mixed powder was heated at 150 °C in an autoclave for 12 h to prepare S/3DOM ZnO composite. The same procedure was repeated with the 3D ZnO to obtain S/3D ZnO composite.

### 2.4. Material Characterizations

The crystal structure of the samples was characterized by X-ray powder diffraction (XRD, Rigaku 3014, Tokyo, Japan). The morphology and architecture of the composites were studied by scanning electron microscope (SEM, JSM-7100F, JEOL, Tokyo, Japan) and transmission electron microscope (TEM, JEM-2100F, JEOL, Tokyo, Japan). The TGA study was performed by TA instruments Q500 (New Castle, DE, USA) at a heating rate of 5 °C min^−1^ under N_2_ atmosphere. The surface area and pore size distribution were obtained by the Brunauer–Emmett–Teller method on a Tristar 3000 instrument (Micromeritics, Norcross, GA, USA). XPS was performed on Thermo Scientific ESCALAB 250Xi (Waltham, MA, USA).

### 2.5. Electrochemical Characterizations

In order to prepare the working electrode, a mixture of 70 wt % active substance, 20 wt % Super-P, and 10 wt % polyvinylidene fluoride (PVDF) was ground adding 1-methyl-2-pyrrolidinone (NMP) solvent to form uniform slurry. Then, the slurry was carefully applied to the current collector and dried overnight in a vacuum oven at 60 °C. The current collector was cut into disks with a diameter of 12 mm. Using Celgard2400 as a separator and Li metal foil as a reference/counter electrode, CR2032-type coin cells were assembled in an Ar-filled glove box. The electrolyte was 1 M LiTFSI in a mixture of 1,3-Dioxolane and 1,2-Dimethoxyethane (DOL and DME) (1:1 in volume) with/without 1 wt % LiNO_3_ additive. The amount of electrolyte used is about 35 μL for electrode with a sulfur loading of 2.0 and 5.0 mg cm^−2^, and the ratio of the amount of electrolyte to the mass of sulfur (E/S) is calculated to be 15.5 and 6.2 mL g^−1^, respectively. Electrochemical tests were carried out with a Land CT2001A battery tester (Land, Wuhan, China) in the potential range of 1.7 to 2.8 V. Cyclic voltammetry (CV) in a potential window of 1.7–2.8 V and electrochemical impedance spectroscopy (EIS) in a frequency range of 0.01–100 kHz were carried out on the CHI760E electrochemical measurement system (CH Instruments, Shanghai, China).

### 2.6. Symmetric Cell Test

The slurry containing 80 wt % 3DOM ZnO (or 3D ZnO), 10 wt % Super P, and 10 wt % PVDF was cast on a carbon cloth to prepare electrodes. Then, symmetric cells were constructed using 0.1 M Li_2_S_6_ in a DOL/DME (1:1 in volume) solution as an electrolyte. The CV and EIS measurements of a symmetrical battery were conducted on a CHI760E workstation.

### 2.7. Linear Sweep Voltammetry Measurement

Using 0.1 M Li_2_S/methanol as an electrolyte and Ag/AgCl as a reference electrode, linear sweep voltammetry (LSV) measurements were carried out with a three-electrode system, and the oxidation behavior of Li_2_S was specifically studied.

### 2.8. Li_2_S Nucleation and Precipitation Test

For this experiment, coin cells were assembled with 3DOM ZnO (or 3D ZnO)-loaded carbon cloth as a working electrode, Li foil as a counter electrode, and Li_2_S_8_/tetramer as an electrolyte. To study the Li_2_S nucleation and growth, a constant current discharge was carried out at 0.112 mA until the cut-off potential, 2.06 V. Then, a constant potential discharge was applied at 2.05 V with the limiting current below 0.01 mA to observe the precipitation phenomenon. 

### 2.9. Absorption Experiment

In order to test the adsorption ability of prepared 3DOM ZnO, a yellow Li_2_S_6_ solution (1 mM) is prepared using Li_2_S and sulfur (1:5) in DME/DOL (1:1 *v*/*v*). Afterwards, 20 mg of 3DOM ZnO or 3D ZnO was added in separate beakers to observe the changes occurred in the solutions.

## 3. Results and Discussion

**Figure 1:**
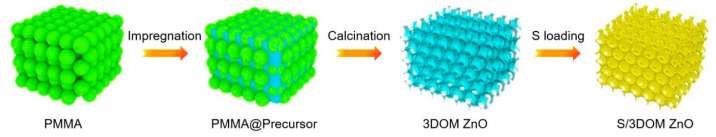
Schematic illustration of the synthesis process of sulfur added to the three-dimensionally ordered macroporous ZnO (S/3DOM ZnO).

Figure 1 shows the synthesis process of S/3DOM ZnO framework. Using PMMA nanospheres as the templates, a 3DOM ZnO skeleton was prepared by impregnation and calcination processes with the subsequent addition of sulfur to form the S/3DOM ZnO composite cathode material.

**Figure 2:**
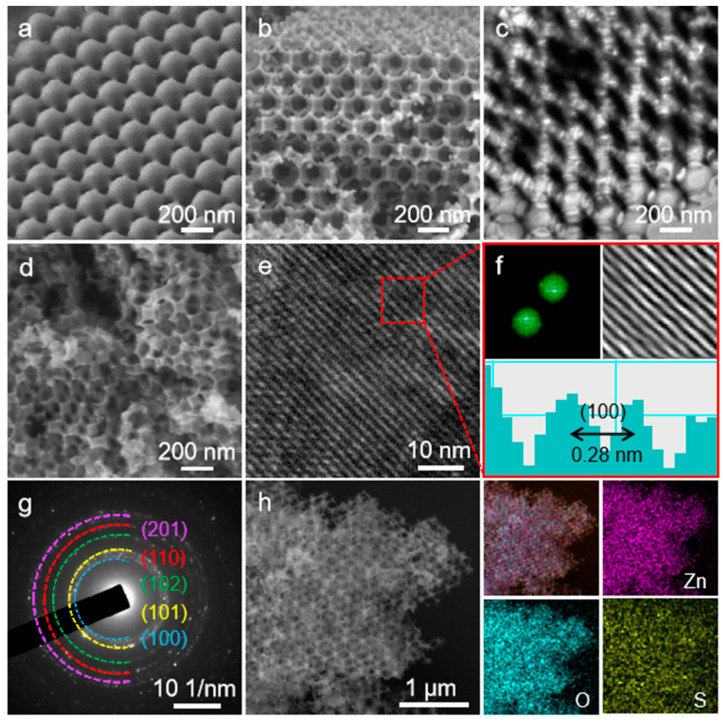
(**a**) SEM image of polymethyl methacrylate (PMMA); (**b**) SEM image and (**c**) TEM image of 3DOM ZnO; (**d**) SEM image of 3D ZnO; (**e**) HRTEM image of 3DOM ZnO; (**f**) FFT pattern and inverse FFT lattice image of 3DOM ZnO; (**g**) SAED patterns of 3DOM ZnO; (**h**) HAADF-STEM image of S/3DOM ZnO and corresponding Zn, O, and S mapping.

Figure 2a shows the perfectly ordered arrangement of PMMA nanospheres with an average size around 200 nm. From the SEM and TEM images (Figure 2b,c) taken from the 3DOM ZnO framework, one can observe that there are many interconnected ordered macropores in the 3DOM ZnO framework, and the average size of the macropores is about 200 nm. Increasing the calcination temperature to 800 °C resulted in destruction of the ordered structure, which leads to the disordered three-dimensional 3D ZnO, as presented in Figure 2d. The High Resolution Transmission Electron Microscope (HRTEM) image of 3DOM ZnO shows that the distance between crystal planes is about 0.28 nm (Figure 2e), corresponding to the crystal plane of ZnO (Figure 2f). Several bright electron diffraction rings were observed in the selected area electron diffraction (SAED) pattern of 3DOM ZnO samples (Figure 2g), indicating the formation of polycrystalline structure [32]. High-Angle Annular Dark Field Scanning Transmission Electron Microscopy (HAADF-STEM) and corresponding elemental mapping images of S/3DOM ZnO composites, depicted in Figure 2h, demonstrate the perfectly ordered three-dimensional structure with the homogenously dispersed sulfur on the surface and in pores of the 3DOM ZnO skeleton.

**Figure 3:**
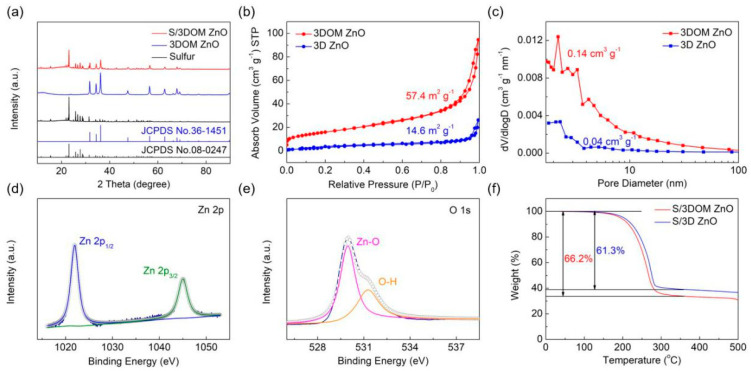
(**a**) XRD patterns of pure sulfur, 3DOM ZnO, and S/3DOM ZnO; (**b**) N_2_ adsorption/desorption isotherms of 3DOM ZnO and 3D ZnO; (**c**) Pore size distribution curves of 3DOM ZnO and 3D ZnO; (**d**) Zn 2p and (**e**) O 1s XPS spectrum of 3DOM ZnO; (**f**) TGA curves of S/3DOM ZnO and S/3D ZnO composites measured under N_2_ atmosphere from room temperature to 600 °C with a heating rate of 10 °C min^−1^.

The XRD pattern of 3DOM ZnO (Figure 3a) matches well with the characteristic peaks of ZnO (JCPDS No. 36-1451), indicating that impurity-free 3DOM ZnO has been successfully synthesized. The peak intensity of sulfur in S/3DOM ZnO composites is obviously weaker than the diffraction peaks from pure sulfur, signifying that sulfur is well dispersed in the pores of the 3DOM ZnO matrix [33]. Figure 3b,c shows the nitrogen adsorption/desorption isotherms and pore size distribution of 3DOM ZnO and 3D ZnO. The surface area and total pore volume of 3DOM ZnO (57.4 m^2^ g^−1^, 0.14 cm^3^ g^−1^) were much higher than those of 3D ZnO (14.6 m^2^ g^−1^, 0.04 cm^3^ g^−1^). These results confirm that the construction of the 3DOM structure can improve the macro/mesoporosity, which is beneficial to the thorough penetration of the electrolyte and rapid ion/mass transport [34]. The XPS spectrum of 3DOM ZnO is shown in Figure 3d,e. From the Zn 2p XPS spectrum, two peaks can be observed at 1021.6 eV and 1044.9 eV, corresponding to Zn 2p_1/2_ and Zn 2p_3/2_, respectively (Figure 3d), confirming the existence of Zn^2+^ [35]. The peaks appeared in O 1s spectrum at 529.8 and 531.4 eV can be ascribed to the Zn-O and O-H bonds, respectively. The content of sulfur in S/3DOM ZnO and S/3D ZnO composites determined by TGA is 66.2% and 61.3%, respectively (Figure 3f), which demonstrates again that the 3DOM ZnO framework is rich in pores, which in turn allows increasing the sulfur loading.

**Figure 4:**
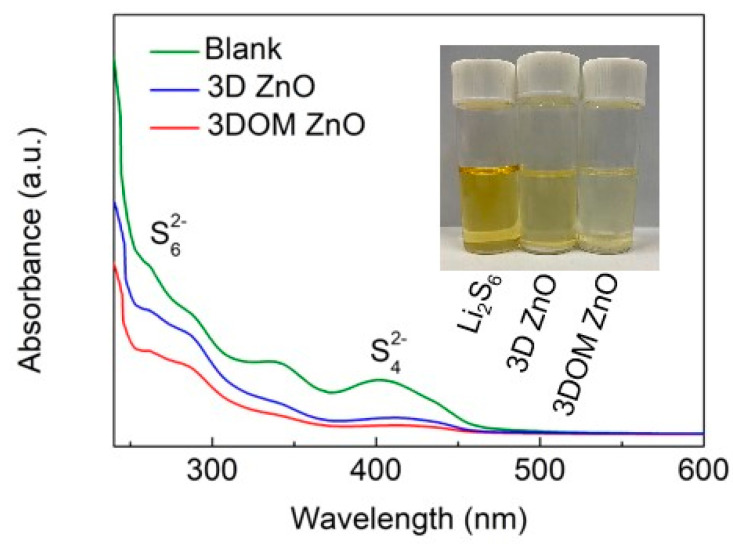
UV-vis spectra and optical images (inset) of Li_2_S_6_ solution before and after adsorption by 3DOM ZnO and 3D ZnO.

The adsorption capacity of 3DOM ZnO was further verified by polysulfide adsorption and UV-vis absorption experiments. As shown in Figure 4 inset, the color of the Li_2_S_6_ solution became slightly lighter with the addition of 3D ZnO. On the contrary, with the addition of 3DOM ZnO, the Li_2_S_6_ solution changed from yellow to almost colorless, meaning that a larger amount of Li_2_S_6_ was adsorbed from the solution by the framework system. The changes in Li_2_S_6_ concentration after adding 3D ZnO and 3DOM ZnO are presented in the UV-vis spectra (Figure 4). The peaks at 270 and 420 nm are related to S_6_^2–^ and S_4_^2–^ species, respectively [36]. The absorption intensity of Li_2_S_6_ solution containing 3DOM ZnO is much weaker than that of Li_2_S_6_ solution containing 3D ZnO. These findings indicate that there is a strong interaction between LiPSs and 3DOM ZnO [37].

**Figure 5:**
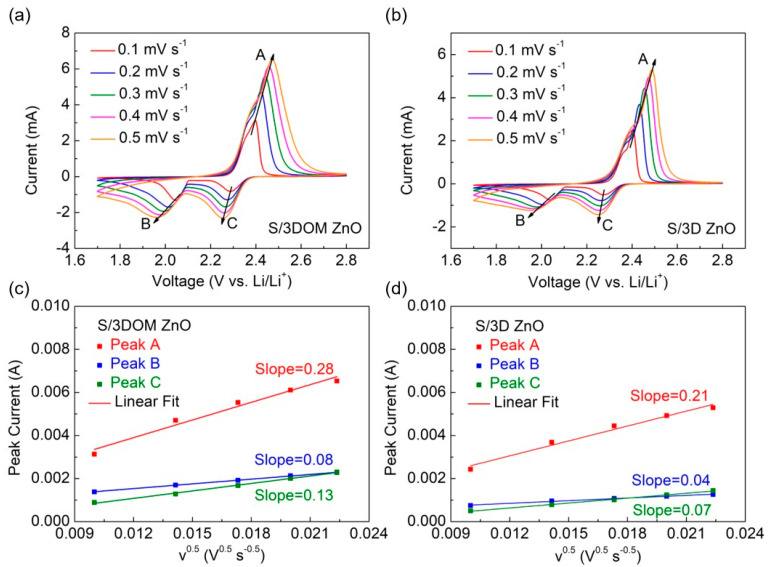
Cyclic voltammetry (CV) curves at various scan rates for (**a**) S/3DOM ZnO and (**b**) S/3D ZnO electrodes; fitting plots of peak current (I_p_) versus the square root of the scan rate (ν^0.5^) for (**c**) S/3DOM ZnO and (**d**) S/3D ZnO electrodes.

The Li^+^ diffusion coefficient is an important parameter to verify the surface diffusion of Li_2_S_x_ and can be estimated with the help of CV. Cyclic voltammograms of S/3DOM ZnO and S/3D ZnO electrodes recorded at different scan rates (0.1–0.5 mV s^−1^) are shown in Figure 5a,b. CV curves of both electrodes have two cathodic peaks and one anodic peak, corresponding to the conversion of S_8_ to Li_2_S_n_ (4 ≤ n ≤ 8), Li_2_S_n_ to Li_2_S_2_/Li_2_S, followed by the reverse reaction with the formation of S_8_, respectively. According to the calculated slope of peak current vs. ν^0.5^, it is clear that S/3DOM ZnO has the higher Li^+^ diffusivity, which further proves that 3DOM ZnO has high catalytic activity (Figure 5c,d) [38].

**Figure 6:**
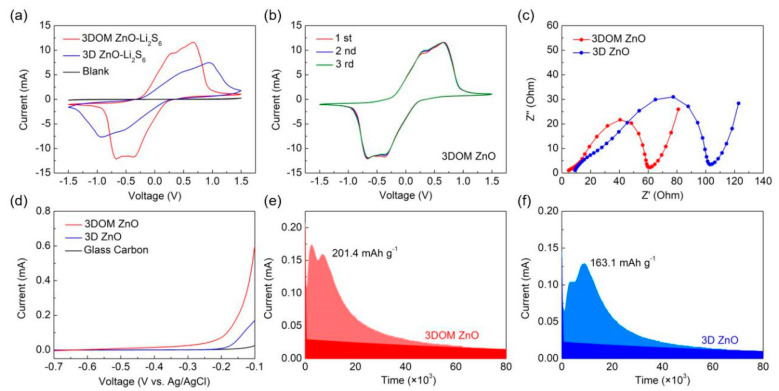
(**a**) CV curves of 3DOM ZnO and 3D ZnO at a scanning rate of 10 mV s^−1^; (**b**) CV curves of 3DOM ZnO at different cycles; (**c**) electrochemical impedance spectroscopy (EIS) spectra of symmetric cells with 3DOM ZnO and 3D ZnO electrodes; (**d**) linear sweep voltammetry (LSV) curves of Li_2_S oxidization for 3DOM ZnO and 3D ZnO electrodes; Li_2_S deposition profiles of (**e**) 3DOM ZnO and (**f**) 3D ZnO.

The enhancement effect of 3DOM ZnO on the redox kinetics of LiPSs was further clarified by the CV experiments. As shown in Figure 6a, the current density of the 3DOM ZnO electrode is significantly higher than that of 3D ZnO electrode, which reflects the rapid redox reaction of 3DOM ZnO. The unique structure of 3DOM promotes faster liquid–solid conversion and shortens the diffusion lifetime of polysulfides, thus suppressing the infamous shuttle effect [39,40]. In addition, the 3DOM ZnO electrode showed good overlapping CV curves in the first three cycles, showing good durability and reversibility (Figure 6b). Figure 6c shows the EIS measurements results of the Li_2_S_6_ symmetrical cell. The semicircle of 3DOM ZnO, which indicates the charge transfer (Rct), is obviously smaller than that of 3D ZnO, indicating that there is an enhanced charge transfer at the interface of 3DOM ZnO.

The oxidation behavior of Li_2_S was studied in detail by the LSV method. As shown in Figure 6d, compared with 3D ZnO and glassy carbon, the initial potential of 3DOM ZnO is the lowest and the current response is the highest, indicating that the activation energy of electrochemical conversion of solid Li_2_S to soluble LiPSs on the surface of 3DOM ZnO is the lowest. In order to further study the promoting effect of 3DOM ZnO on the liquid–solid conversion, the constant potential Li_2_S precipitation experiment was designed. As shown in Figure 6e,f, the precipitation capacity of Li_2_S on the 3DOM ZnO surface (201.4 mAh g^−1^) is larger than that on the 3D ZnO surface (163.1 mAh g^−1^). The results showed that the 3DOM ZnO electrode significantly reduced the nucleation overpotential of Li_2_S and promoted the precipitation of Li_2_S [41].

**Figure 7:**
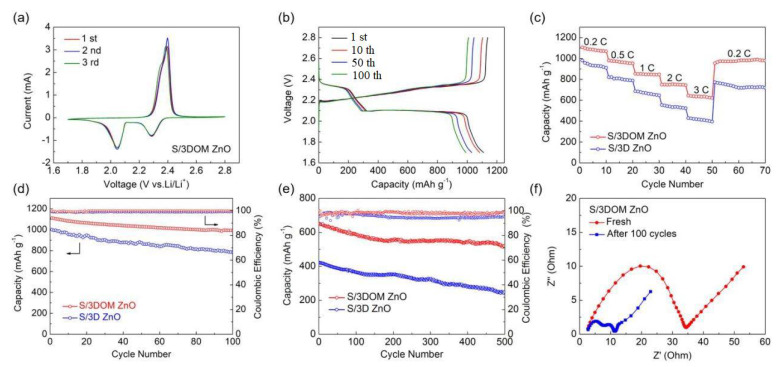
(**a**) CV curves of S/3DOM ZnO at 0.1 mV s^−1^; (**b**) galvanostatic charge/discharge profiles of S/3DOM ZnO electrode at 0.2 C; (**c**) rate performance of S/3DOM ZnO and S/3D ZnO electrodes at different C-rates; (**d**) cycling performance at 0.2 C and (**e**) long-term cycling performance at 3 C of S/3DOM ZnO and S/3D ZnO electrodes; (**f**) Nyquist plots of S/3DOM ZnO electrodes before and after 100 cycles.

Figure 7a shows the first three cyclic voltammetry curves of S/3DOM ZnO scanned at 0.1 mV s^−1^ within a potential window of 1.7–2.8 V. Two reduction peaks were observed near 2.05 and 2.3 V, and an oxidation peak was observed at about 2.4 V. Only a small shift of the peak positions was recorded after three cycles, which means that the redox process of S/3DOM ZnO is highly reversible. Figure 7b shows the charge–discharge potential profiles of the S/3DOM ZnO electrode during the cycles. The discharge curve has two distinct plateaus at 2.1 V and 2.3 V, while the charge curve demonstrates a rising plateau at around 2.4 V. Defined potentials where the plateaus appear are consistent with the redox potentials in the CV curves. Even after 100 cycles, all plateaus remain stable, meaning that unique characteristics of 3DOM ZnO can effectively inhibit the shuttle of the polysulfides.

Rate capabilities of the S/3D ZnO and S/3DOM ZnO recorded at various C-rates from 0.2 C to 3.0 C are depicted in Figure 7. The discharge capacities of the S/3DOM ZnO electrode at 0.2, 0.5, 1.0, 2.0, and 3.0 C are estimated to be 1105, 987, 862, 753, and 651 mAh g^−1^, respectively (Figure 7c). When the rate is restored to 0.2 C after a high C-rate, the capacity of 954 mAh g^−1^ is obtained. In contrast, the rate performance of the S/3D ZnO electrode is significantly lower than that of the S/3DOM ZnO electrode, indicating that the mesoporous network framework in 3DOM facilitates a fast mas/ion transfer. The cycle performance of the S/3D ZnO and S/3DOM ZnO electrodes over 100 cycles at 0.2 C and over 500 cycles at 3 C are shown in Figure 7d,e. The capacity of the S/3DOM ZnO electrode reaches 1110 mAh g^−1^ and still maintains 991 mAh g^−1^ after 100 cycles, i.e., it exhibits a high capacity retention of 89.3%. As shown in Figure 7e, a capacity of 512 mAh g^−1^ after 500 cycles at 3 C (a decay rate of 0.043%) can be obtained for the S/3DOM ZnO electrode, which is much higher than that of S/3D ZnO electrode. More importantly, the coulombic efficiency of the S/3DOM ZnO electrode is almost 99%. The results show that 3DOM ZnO not only inhibits the shuttle effect through strong chemical and physical interactions with polysulfides, but it also promotes the redox kinetics of polysulfides by a catalytic electrochemical reaction. The SEM image after cycling is shown in Figure S1. The well-defined 3D ordered porous architecture can be maintained after cycling, indicating the good structural integrity of 3DOM ZnO upon the battery operation.

In general, when using the electrolyte without LiNO_3_, no stable SEI layer can be formed on the surface of the lithium anode, so the shuttled polysulfides reacts with lithium metal continuously, and the formation of insulating Li_2_S on the lithium anode will lead to irreversible capacity loss [42]. In order to demonstrate the advantages of reactivity of 3DOM ZnO toward polysulfides in such conditions, the cycling performance of a Li-S cell free of LiNO_3_ was evaluated. As shown in Figure S2, a Li-S battery free of LiNO_3_ still maintains a capacity of 751 mAh g^−1^ after 100 cycles, i.e., 77.5% of its initial capacity. These results further demonstrate that the 3DOM ZnO is an effective sulfur host capable of improving the cycling performance of Li-S batteries and effectively restraining the shuttle effect of polysulfides. In addition, in order to evaluate the performance of a higher energy density S/3DOM ZnO electrode, the cycling performance of the electrode with high sulfur loading was studied, and the results of these tests are shown in Figure S3. The S/3DOM ZnO electrode delivers a high initial areal capacity of 4.47 mAh cm^−2^ and could maintain a capacity of 3.19 mAh cm^−2^ after 50 cycles. Such enhanced capacity retention can be attributed to the structural and chemical advantages of 3DOM ZnO, which provide efficient sulfur dissolution and diffusion restrictions and support fast redox reaction.

The results of EIS analysis for the S/3DOM ZnO electrode before and after 100 cycles are shown in Figure 7f. The Nyquist plots consist of a semicircle in the high-frequency region, which corresponds to the charge transfer resistance (R_ct_) and an inclined line in the low-frequency region, which refers to the solid-state diffusion resistance (W_s_) [43]. The S/3DOM ZnO electrode possesses a small semicircle diameter and high slope, indicating a low charge transfer resistance value and fast diffusion rates. After 100 cycles, the R_ct_ value of the S/3DOM ZnO electrode is less than the initial value due to the dissolution and redistribution of sulfur [44].

The comparative data of the electrochemical performance of S/3DOM ZnO electrode with the previously reported S/C electrodes are shown in Table S1. It is worth noting that considering the composition and similar testing conditions, the S/3DOM ZnO electrode exhibits enhanced cycling performance and higher capacity retention, which is due to its capability of effective physical and chemical restrictions of the polysulfides diffusion and fast sulfur conversion kinetics during long-term cycling. However, the initial discharge capacity and rate performance of S/3DOM ZnO electrode is slightly lower than those of some of the carbon-based electrodes, which could be attributed to a lower conductivity of ZnO.

## 4. Conclusions

In summary, a 3DOM ZnO framework was prepared by a template method as an advanced sulfur host for lithium–sulfur batteries. The macroporous framework not only facilitates faster and better penetration of the electrolyte accelerating mass/charge transfer, but it also can evenly distribute sulfur and inhibit its dissolution, thus improving the utilization of sulfur. The exposed large active surface area of ZnO provides a strong chemical interaction with LiPSs, and it plays a useful catalytic role in the transformation of LiPSs, which can obviously inhibit the shuttle of polysulfides and promote the kinetics of sulfur redox reaction. Therefore, the S/3DOM ZnO electrode delivers a high capacity of 1110 mAh g^−1^ at 0.2 C and excellent cycle stability with low capacity decay (0.043%) over 500 cycles at 3 C, as well as high areal capacity and decent cyclic stability under high sulfur loading. The successful design of 3DOM ZnO provides a new avenue to construct advanced sulfur electrodes.

## Supplementary Materials

The following are available online at https://www.mdpi.com/2079-4991/10/11/2267/s1, Figure S1: SEM image of S/3DOM ZnO after 50 cycles, Figure S2: Cycling performance of the Li-S battery with S/3DOM ZnO electrode free of LiNO_3_, Figure S3: Cycling performance of S/3DOM ZnO electrode at 0.2 C under high sulfur loading (5 mg cm^–2^), Table S1: The comparison of the electrochemical performance of S/3DOM ZnO electrode with the previously reported S/C electrodes.
