# Supplementary material for: Three-Dimensionally Ordered Macroporous ZnO Framework as Dual-Functional Sulfur Host for High-Efficiency Lithium–Sulfur Batteries
Source: Nanomaterials (Basel). 2020 Nov 16;10(11):2267. doi: 10.3390/nano10112267 (PMC7697050; doi:10.3390/nano10112267)
Supplement: Supplementary file 1 [file nanomaterials-10-02267-s001.pdf]

Supplementary Materials

Three-Dimensionally Ordered Macroporous ZnO Framework as Dual-Functional Sulfur Host for High-Efficiency Lithium–Sulfur Batteries

Haisheng Han [1,2†], Tong Wang [1,2†], Yongguang Zhang [1,2*], Arailym Nurpeissova [3] and Zhumabay Bakenov [3]

[1] School of Materials Science and Engineering, Hebei University of Technology, Tianjin 300130, China; 15022610664@139.com (H.H.); 13720007220@139.com (T.W.)

[2] Tianjin Key Laboratory of Materials Laminating Fabrication and Interface Control Technology, Hebei University of Technology, Tianjin 300130, China

[3] Department of Chemical and Materials Engineering, National Laboratory Astana, Nazarbayev University, Nur-Sultan 010000, Kazakhstan; arailym.nurpeissova@nu.edu.kz (A.N.); zbakenov@nu.edu.kz (Z.B.)

* Correspondence: yongguangzhang@hebut.edu.cn

† The authors contributed equally to this work.

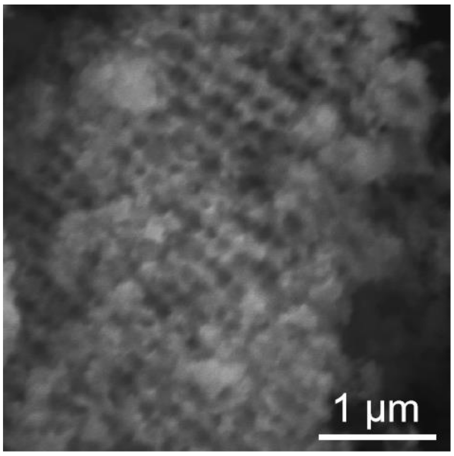

Figure S1. SEM image of S/3DOM ZnO after 50 cycles.

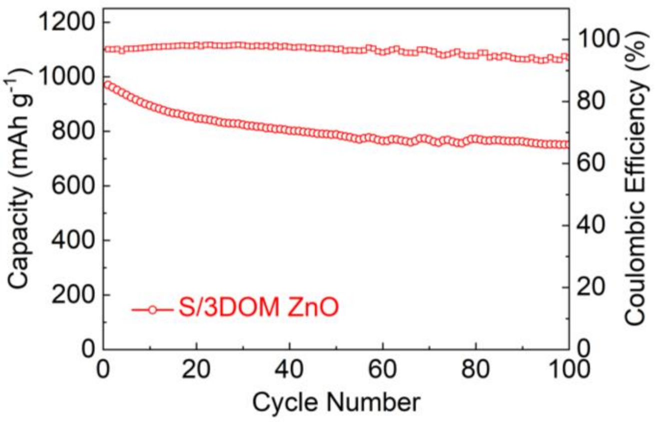

Figure S2. Cycling performance of the Li-S battery with S/3DOM ZnO electrode free of $LiNO_3$.

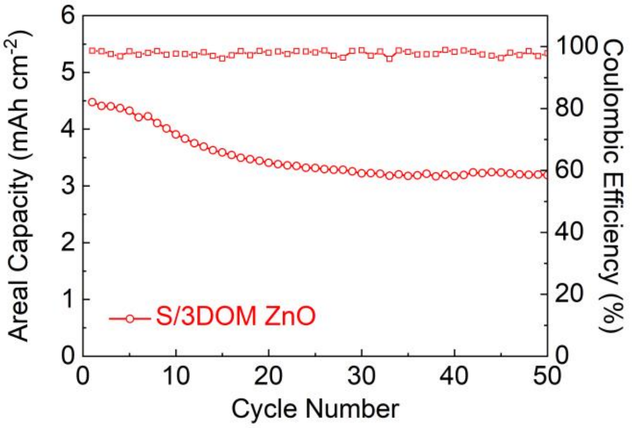

Figure S3. Cycling performance of S/3DOM ZnO electrode at 0.2 C under high sulfur loading (5 mg cm^{-2}).

Table S1. The comparison of the electrochemical performance of S/3DOM ZnO electrode with the previously reported S/C electrodes.

Electrode material	Sulfur Loading	Initial capacity (mAh g^{-1}, at n C)	Capacity retention (%, at n C, after n Cycle)	High Rate Performance (mAh g^{-1}, at n C)	Ref.
HPC-S	1 mg cm^{-2}	998 mAh g^{-1} (0.3 C)	77% (2.4 C, 500 th)	700 mAh g^{-1} (2.4 C)	[1]
3D S@PGC	2.36 mg cm^{-2}	1382 mAh g^{-1} (0.5 C)	61% (2 C, 1000 th)	500 mAh g^{-1} (5 C)	[2]
3D/S/VCNs	1.0–1.5 mg cm^{-2}	1240 mAh g^{-1} (167 mA g^{-1})	80.3% (837 mA g^{-1}, 300 th)	738 mAh g^{-1} (3340 mA g^{-1})	[3]
S/3DOM-m C	2 mg cm^{-2}	1042 mAh g^{-1} (0.2 C)	67.4% (0.2 C, 100 th)	357 mAh g^{-1} (2.5 C)	[4]
S/C-1.5	1.0–1.5 mg cm^{-2}	870 mAh g^{-1} (0.5 C)	85% (1 C, 300 th)	703 mAh g^{-1} (2 C)	[5]
PCKH/S	1.2 mg cm^{-2}	1188.6 mAh g^{-1} (0.1 C)	60% (1 C, 300 th)	668.1 mAh g^{-1} (2 C)	[6]
S/3DOM ZnO	2 mg cm^{-2}	1110 mAh g^{-1} (0.2 C)	78.6% (3 C, 500 th)	651 mAh g^{-1} (3 C)	This work

References

[1] Jung, D.; Hwang, T.; Lee, J.; Koo, H.; Shakoor, R.A.; Kahraman, R.; Jo, Y.; Park, M.S.; Choi, J. Hierarchical Porous Carbon by Ultrasonic Spray Pyrolysis Yields Stable Cycling in Lithium-Sulfur Battery. *Nano Lett.* **2014**, *14*, 4418–4425.

[2] Li, G.; Sun, Ji.; Hou, W.; Jiang, S.; Huang, Y.; Geng, J. Three-dimensional porous carbon composites containing high sulfur nanoparticle content for high-performance lithium-sulfur batteries. *Nat. Commun.* **2016**, *7*, 10601.

[3] Rehman, S.; Gu, X.; Khan, K.; Mahmood, N.; Yang, W.; Huang, X.; Guo, S.; Hou, Y. 3D Vertically Aligned and Interconnected Porous Carbon Nanosheets as Sulfur Immobilizers for High Performance Lithium-Sulfur Batteries. *Adv. Energy Mater.* **2016**, *6*, 1502518.

[4] Zhang, C.; Zhang, Z.; Wang, D.; Yin, F.; Zhang, Y. Three-dimensionally ordered macro-/mesoporous carbon loading sulfur as high-performance cathodes for lithium/sulfur batteries. *J. Alloys Compd.* **2017**, *714*, 126–132.

[5] Díez, N.; Ferrero, G.A.; Sevilla, M.; Fuertes, A.B. A simple and general approach for in situ synthesis of sulfur-porous carbon composites for lithium-sulfur batteries. *Sustainable Energy Fuels* **2019**, *3*, 3498–3509.

[6] Xiao, Q.; Li, G.; Li, M.; Liu, R.; Li, H.; Ren, P.; Dong, Y.; Feng, M.; Chen, Z. Biomass-derived nitrogen-doped hierarchical porous carbon as efficient sulfur host for lithium-sulfur batteries. *J. Energy Chem.* **2020**, *44*, 61–67.
